# Renin–Angiotensin System: An Important Player in the Pathogenesis of Acute Respiratory Distress Syndrome

**DOI:** 10.3390/ijms21218038

**Published:** 2020-10-28

**Authors:** Jaroslav Hrenak, Fedor Simko

**Affiliations:** 1 Department of Cardiovascular Surgery, Inselspital – University Hospital of Bern, Freiburgstrasse 18, 3010 Bern, Switzerland; jaroslav.hrenak@insel.ch; 2 Institute of Pathophysiology, Faculty of Medicine, Comenius University, Sasinkova 4, 811 08 Bratislava, Slovak; 3 3rd Department of Internal Medicine, Faculty of Medicine, Comenius University, Limbova 5, 833 05 Bratislava, Slovak; 4 Institute of Experimental Endocrinology, Biomedical Research Center, Slovak Academy of Sciences, Dúbravská cesta 9, 845 05 Bratislava, Slovak

**Keywords:** ARDS, renin–angiotensin system, ACE2, COVID-19, SARS-CoV-2

## Abstract

Acute respiratory distress syndrome (ARDS) is characterized by massive inflammation, increased vascular permeability and pulmonary edema. Mortality due to ARDS remains very high and even in the case of survival, acute lung injury can lead to pulmonary fibrosis. The renin–angiotensin system (RAS) plays a significant role in these processes. The activities of RAS molecules are subject to dynamic changes in response to an injury. Initially, increased levels of angiotensin (Ang) II and des-Arg^9^-bradykinin (DABK), are necessary for an effective defense. Later, augmented angiotensin converting enzyme (ACE) 2 activity supposedly helps to attenuate inflammation. Appropriate ACE2 activity might be decisive in preventing immune-induced damage and ensuring tissue repair. ACE2 has been identified as a common target for different pathogens. Some Coronaviruses, including SARS-CoV-2, also use ACE2 to infiltrate the cells. A number of questions remain unresolved. The importance of ACE2 shedding, associated with the release of soluble ACE2 and ADAM17-mediated activation of tumor necrosis factor-α (TNF-α)-signaling is unclear. The roles of other non-classical RAS-associated molecules, e.g., alamandine, Ang A or Ang 1–9, also deserve attention. In addition, the impact of established RAS-inhibiting drugs on the pulmonary RAS is to be elucidated. The unfavorable prognosis of ARDS and the lack of effective treatment urge the search for novel therapeutic strategies. In the context of the ongoing SARS-CoV-2 pandemic and considering the involvement of humoral disbalance in the pathogenesis of ARDS, targeting the renin–angiotensin system and reducing the pathogen’s cell entry could be a promising therapeutic strategy in the struggle against COVID-19.

## 1. Introduction

Acute respiratory distress syndrome (ARDS) is defined as an acute, diffuse, inflammatory lung injury, leading to increased pulmonary vascular permeability, increased lung weight, and loss of aerated lung tissue [1]. An efficient pharmacological treatment is lacking and mechanical lung ventilation or even extracorporeal membrane oxygenation is needed to ensure sufficient gas exchange. Despite great progress in this field, overall mortality is 45% [2]. Moreover, mechanical ventilation itself can contribute to lung injury. The prevalence of ventilator-associated lung injury is 6.2% among mechanically ventilated patients [3,4]. The most common risk factor for developing ARDS is pneumonia (35–50%), followed by non-pulmonary sepsis (30%), aspiration (10%), and trauma (10%) [5]. The pathogenesis of ARDS involves diffuse alveolar damage, inflammation with a cytokine storm and increased capillary permeability, resulting in the development of pulmonary edema, decreased lung compliance and impaired gas exchange [6]. The reabsorption of alveolar and interstitial fluid is associated with the proliferation of type II alveolar cells as well as fibroblasts and myofibroblasts producing components of the extracellular matrix. These processes can lead either to the restoration of normal pulmonary tissue or to fibrotic remodeling of the lungs [7]. The underlying mechanisms determining the loss of lung parenchyma and fibrosis development are still insufficiently understood.

**Figure 1:**
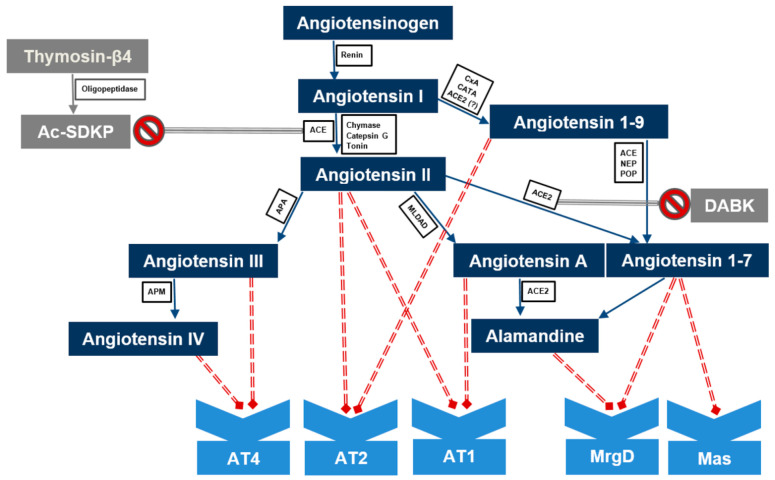
Renin–angiotensin system. ACE—angiotensin-converting enzyme; ACE2—angiotensin-converting enzyme 2. Ac-SDKP—N-acetyl-seryl-aspartyl-lysyl-proline; DABK—des-Arg^9^-bradykinin; CxA—carboxypeptidase A; CATA—cathepsin A; NEP—neprilysin; POP—propyl oligopeptidase; APA—aminopeptidase A; MLDAD—mononuclear leukocyte-derived aspartate decarboxylase; APM—aminopeptidase M, AT1, AT2, AT3 angiotensin receptor type 1, 2, 3, respectively; MrgD— Mas-Related G-Protein Coupled Receptor D; Mas—Mas receptor.

The renin–angiotensin system (RAS) (Figure 1) represents one of the most important regulatory mechanisms in both physiological and pathological conditions [8,9]. Its components are present in various tissues and are involved in a number of biological pathways [8,10,11]. The RAS has been known for over 120 years and has been mostly studied in relation to blood pressure regulation and cardiovascular disorders [12,13,14,15]. In addition to their hemodynamic effects, components of the RAS act in various organs and systems as signaling molecules modulating inflammation, oxidative stress, cell proliferation, tissue remodeling, and apoptotic or necrotic cell death [15,16,17,18,19].

In the last three decades, several other “non-classical” RAS peptides such as the type 2 angiotensin II receptor (AT2R) [20,21], the angiotensin converting enzyme (ACE) 2 [22], angiotensin (Ang) 1–7 [23], alamandine and Ang A [11] or Ang 1–9 [24] have been identified. Recent data suggest that they are more than inactive splitting products. They are involved in various reactions at the cellular and organ levels [8,9]. Nowadays, the concept of two counterbalancing axes is generally accepted. The classical cascade, represented by ACE/Ang II/AT1R, exerts vasoconstrictor, pro-inflammatory and profibrotic effects. The other, involving ACE2, Ang 1–7 and the Mas receptor (MasR), mediates the vasodilatory, anti-inflammatory and anti-fibrotic actions of the RAS. However, the interactions of the RAS family members are multidirectional, involving a bulk of molecules whose physiological role has yet to be elucidated [8,11,25]. In addition, the RAS is closely related to the kinin–kalikrein system, regulating vascular tone and permeability, since ACE and ACE2 participate in the inactivation of bradykinin and des-Arg^9^-bradykinin (DABK), respectively [26,27].

## 2. RAS and Pulmonary Diseases

An upregulation of Ang II/AT1R-mediated signaling has been identified in various pathologic conditions, including pulmonary diseases [18,28]. Indeed, the activation of the ACE/Ang II/AT1R cascade is associated with increased pneumonia risk and worse prognosis [29]. In experimental lipopolysaccharide (LPS)-induced lung fibrosis, Ang II levels were slightly elevated in the plasma and even more increased in the bronchoalveolar lavage fluid. These changes were accompanied by higher AT1R expression in the pulmonary tissue [30]. Similarly, increased Ang II levels were also detected in the serum of patients with avian influenza H5N1 [31]. ARDS non-survivors were reported to have had significantly higher Ang I levels [32]. Patients with influenza (H7N9) discharged from hospital within 28 days presented a decline in Ang II plasma levels after the first week, whereas they remained elevated in patients who required longer hospitalization and in non-survivors [33].

On the other hand, stimulation of the Ang 1–7/ACE2 axis seems to be beneficial in acute lung injury. Treatment with human recombinant ACE2 attenuated bleomycin-induced fibrosis and improved pulmonary function in ACE2 wild-type mice [34]. Intravenous administration of ACE2 also attenuated the LPS-induced overexpression of pro-inflammatory cytokines interleukin (IL)-1β, IL-6 and tumor necrosis factor α (TNF-α) [35]. In mice infected with H5N1 influenza virus, treatment with recombinant ACE2 reduced the virus replication in the lungs and even reduced mortality [31]. Similarly, the administration of Ang 1–7 in rats subjected to acid aspiration and high stretch ventilation reduced the number of inflammatory cells in bronchoalveolar lavage and attenuated pulmonary fibrosis two weeks after the acid aspiration injury [36]. The beneficial effects of Ang 1–7 administration in LPS-induced lung injury were related to a downregulation of AT1R [30].

## 3. Regulatory, Rather than Harmful or Protective

Generally, the two principal RAS pathways—ACE/Ang II/AT1R and ACE2/Ang 1–7/MasR—are designated as deleterious and protective, respectively. However, these two cohorts of apparently counterregulatory molecules are more likely to be involved in complex and sophisticated regulatory processes and their positive or negative impact depends on a wide variety of factors.

**Figure 2:**
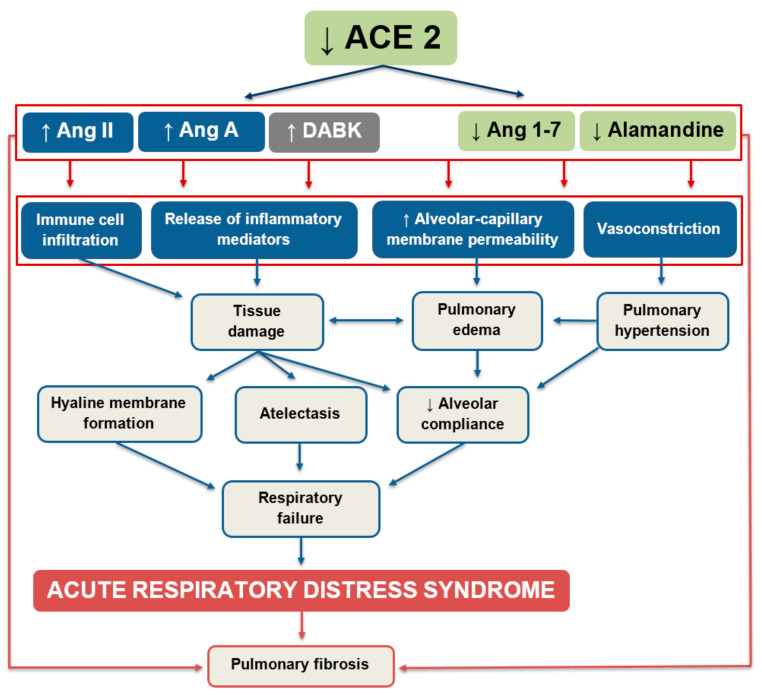
Putative role of the renin–angiotensin system in the progression of acute lung injury. ACE2—angiotensin-converting enzyme 2; Ang II—angiotensin II; Ang A—angiotensin A; DABK—des-Arg^9^-bradykinin; Ang 1–7—angiotensin 1–7.

An overwhelming activity of the pro-inflammatory Ang II-mediated cascade in relation to the ACE2/Ang 1–7 pathway seems to be necessary for an adequate immune response to respiratory infections. Lower ACE2 levels stimulate neutrophil accumulation in the lungs, which is an important step in defending against microbial invasion [37,38]. Besides Ang II and Ang A, ACE2 cleaves another pro-inflammatory mediator, DABK, in the respiratory system [27]. Attenuated ACE2 activity in response to bacterial infection leads to decreased inactivation and increased bioavailability of DABK, resulting in the release of pro-inflammatory mediators such as C-X-C motif chemokine 5 (CXCL5), macrophage inflammatory protein-2 (MIP2), C-X-C motif chemokine 1 (KC), and tumor necrosis factor (TNF)-α from airway epithelia, and increased neutrophil infiltration [27]. Thus, in the early stages of a bacterial infection, the dynamic balance within the RAS is prone to be shifted towards Ang II and DABK to recruit immunocompetent cells to the lungs. Later, ACE2 activity increases to avoid excessive inflammation and cytokine-related tissue damage. The existence of both axes within the RAS seems to be unavoidable for the proper regulation of the immune response, which requires dynamic changes in the activities of the RAS-associated compounds in the course of the inflammatory reaction. ACE/Ang II/AT1R signaling thus becomes deleterious in the case of disrupted equilibrium within the RAS (Figure 2).

## 4. ACE2: Conductor of Inflammatory Response in the Lungs

**Figure 3:**
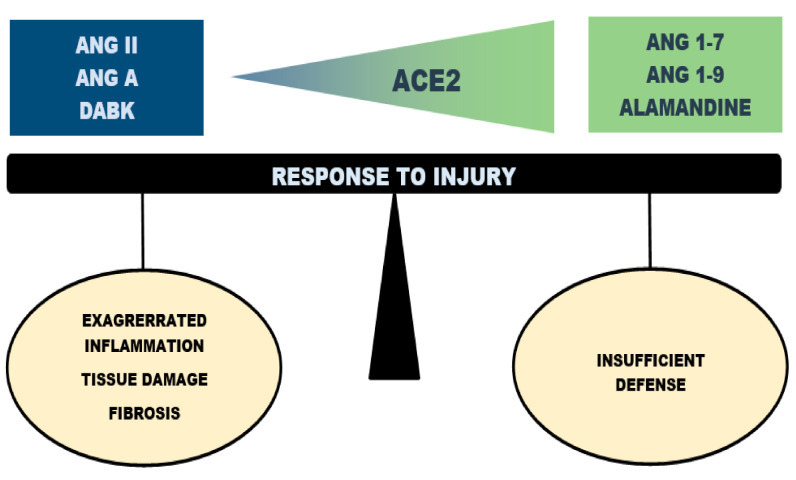
Regulatory role of angiotensin-converting enzyme 2 in the pathogenesis of acute lung injury and ARDS. Increasing/decreasing activity of the angiotensin-converting enzyme 2 (ACE2) regulates the dynamic balance within the RAS. Ang II—angiotensin II; Ang A—angiotensin A; DABK—des-Arg^9^-bradykinin; Ang 1–7—angiotensin 1–7; Ang 1–9—angiotensin 1–9.

ACE2, a carboxypeptidase identified in 2000 as a human homologue of ACE, is apparently a key molecule regulating inflammation in the respiratory system (Figure 3). ACE2 exhibits approximately 40% identity and 61% similarity compared to ACE [22]. Due to the slight structural difference between ACE and ACE2, ACE inhibitors do not inhibit ACE2 activity [39]. Several natural substrates for ACE2 have been identified. The best-studied pathway is the conversion of Ang II to generate Ang 1–7 [23]. ACE2 also further metabolizes Ang A to alamandine [11] as well as other peptides, including DABK, apelin, neurotensin, dynorphin, ghrelin [8,22,27,40,41,42,43]. The formation of Ang 1–9 from Ang I has also been reported; however, its role in vivo is disputable since the kinetic of the reaction is rather poor [39,42].

High ACE2 gene expression has been found in the heart, kidney, brain, lungs, oral mucosa, liver and small intestine [22,43,44,45,46,47]. A recent analysis of human tissues showed that ACE2 was predominantly expressed on the barrier sides of bronchial or skin cells and was more abundant in the bronchial biopsies of smokers [48]. ACE2 occurs in membrane-bound (mACE2) and soluble (sACE2) forms. The ectodomain of mACE2 is cleaved from the cell membrane and subsequently released as sACE2 into the interstitial space and circulation [49,50]. This splitting happens in a constitutive or inducible way [49,51,52], by the enzymatic action of the tumor necrosis factor-α (TNF-α)-converting enzyme (TACE), also known as ADAM17 [50], or other sheddases [51]. Importantly, sACE2 was shown to maintain its catalytic activity [49].

ADAM17-dependent ACE2 shedding can be induced by Ang II via AT1R as well as by elevated glucose levels or by several members of the Coronavirus family, including SARS-CoV and SARS-CoV-2 [52,53,54,55,56,57]. Higher sACE2 plasma levels were observed in men compared to women, the older population, post-menopausal women and in patients with metabolic syndrome or heart failure [58,59,60]. The role of ACE2 shedding under physiological and pathological conditions remains, however, insufficiently understood. Non-sheddable ACE2 exerted impaired enzymatic activity, indicating that attenuated ACE2 shedding might be involved in the pathogenesis of several diseases [18]. On the other hand, enhanced ACE2 shedding in the brain was shown to contribute to the pathogenesis of deoxycorticosterone acetate (DOCA)-salt-induced hypertension in mice [61].

ADAM17 catalyzes, among others, the activation of pro-TNF-α to TNF-α [50]. Increased ADAM17 activity was detected in several pathologies, such as diabetes mellitus, hypertension, inflammatory processes, cardiovascular disorders, neurological and oncological diseases [50,61]. A knockdown of ADAM17 in the DOCA-salt hypertensive model prevented the decline in ACE2 in the brain and attenuated hypertension, whereas an overexpression of ACE2 was associated with reduced ADAM17 levels [61].

Importantly, the ADAM17-mediated pathway is not the only mechanism to cleave ACE2 from the cell surface [18,51]. The inhibition of ADAM17 in proximal tubular cells in mice did not abolish the constitutive ACE2 shedding [52,53] but it was inhibited by blocking protein kinase C-δ (PKC-δ) [53]. In addition, under experimental conditions, at least two forms of sACE2, referred to as the large and small soluble forms, differing in their molecular masses, have been identified, while ADAM17 is only involved in the generation of the large soluble form of ACE2 [51].

## 5. Interactions with Infectious Agents

Considering the fact that infectious diseases, particularly pneumonia and sepsis, account for the greatest number of ARDS cases [5], understanding the mutual interactions between the RAS and microbial agents is of significant value.

Attenuated ACE2 activity was observed in LPS-induced pneumonia, which represents a well-established experimental model of bacterial respiratory infection. Although LPS enhanced ACE2 gene expression, transcriptional and posttranscriptional modifications led to its reduced bioavailability in mouse lungs [27]. The influenza virus also downregulates ACE2, most likely due to the direct catalytic action of virus surface protein neuraminidase, followed by further protein degradation by the proteasome pathway [62]. ACE2 degradation has no impact on the replication of the influenza virus [62,63]; however, it is conceivable that lower ACE2 bioavailability can affect the immune response to the infection and facilitate the progression of the disease.

The SARS epidemic in 2003 as well the current COVID-19 pandemic, caused by two members of the Coronavirus family SARS-CoV and SARS-CoV-2, respectively, have directed attention towards the interaction of Coronaviruses with the RAS. mACE2 serves as a binding site for SARS-CoV [64,65] and SARS-CoV-2 [66,67], mediating the entry of the virus into the host cell. A mutation in the receptor-binding domain of the SARS-CoV-2 spike protein S compared to SARS-CoV is responsible for a higher affinity of SARS-CoV-2 to ACE2 [67].

mACE2, among others, is highly expressed by type II alveolar cells, facilitating virus entry and replication in these cells [68]. The internalization of the virus into the host cells requires a priming of the spike protein S after binding to mACE2, which is associated with cleavage at the S1/S2 and the S2 sites by transmembrane protease serine 2 (TMPRSS2) [69]. ACE2 and TMPRSS2 co-expression on type II alveolar cells or nasal epithelial cells facilitates the infection of the host cells [69,70]. Interaction with the SARS-CoV spike protein induces the downregulation of ACE2 [65,71], preventing co-infection by other intracellular pathogens [56,71]. The binding of the SARS-CoV spike protein to ACE2 led to attenuated ACE2 protein expression in vivo as well as to a reduced number of mACE2 in Vero E6 cells [65]. Another mechanism contributing to the loss of mACE2 is the ADAM17-dependent shedding of ACE2 [56]. Importantly, only mACE2 can mediate virus entry into the cell [49]. However, released sACE2 retains the ability to bind the virus and inhibit its internalization into the cells [49,72].

Another human Coronavirus (HCoV)-NL63, causing the common cold, also attenuates ACE2 expression in infected cells, while this effect is proportional to virus replication efficacy [73]. However, the spike protein of HCoV-NL63 has lower affinity to ACE2 compared to SARS-CoV, which determines its lower virulence [71].

The ways in which different microorganisms interfere with the RAS are probably agent-specific, while ACE2 downregulation seems to be the common feature. Attenuated ACE2 activity presumably facilitates prolonged and more intense activation of the pro-inflammatory RAS-associated pathways, leading to immune-induced tissue damage and fibrosis development in the later course of the disease.

## 6. Pharmacological Inhibition of ACE and AT1R in Pulmonary Diseases

ACE inhibitors (ACEIs) or AT1R blockers (ARBs) are well established in the treatment of hypertension, heart failure, renal pathologies and the prevention of atherosclerotic complications. Considering the role of the RAS in the pathogenesis of pulmonary diseases, pharmacological antagonists of the RAS could modulate the evolution of lung injury by interfering with local RAS in the lungs (Table 1).

An in vitro assay on LPS-treated isolated cells showed that the administration of the ACEI enalapril, ARB valsartan or both alleviated LPS-induced pulmonary inflammation [35]. In clinical settings, a number of data indicate the potential benefit of ACEIs/ARBs in pulmonary inflammatory diseases. A large population-based cohort study involving more than 250,000 Canadian patients 65 years of age or older documented a reduced number of hospitalizations with pneumonia in the first 90 days after the initiation of antihypertensive therapy in favor of ACEIs or ARBs compared to other antihypertensive drugs [74]. A smaller Korean study showed reduced pneumonia risk in elderly patients (aged <70 years) with chronic obstructive pulmonary disease (COPD) treated with ACEIs or ARBs [75]. A retrospective analysis of 215,225 American patients revealed that treatment with ACEIs or ARBs slowed the progression of pulmonary complications in COPD patients [76]. The analysis of the medical records of 182 ARDS patients showed an increased survival rate in patients treated with ACEIs or ARBs [77]. The Cox regression analysis of the data obtained in the longitudinal observational Fremantle Diabetes Study Phase II, found that the use of ACEIs/ARBs was associated with a reduced risk of pneumonia/influenza in patients with type 2 diabetes mellitus [78].

## 7. ACEIs and ARBs in COVID-19

The global COVID-19 pandemic has raised concerns regarding the impact of ACEIs and ARBs on susceptibility to SARS-CoV-2 and the severity of the infection. Since the virus uses mACE2 to enter into the host cells, some authors have hypothesized that increased ACE2 bioavailability related to treatment with ACEIs or ARBs might facilitate the entry of SARS-CoV-2 into the cells [80,81]. However, the current evidence does not support this opinion [82]. A meta-analysis of nine studies including 3936 patients with hypertension and COVID-19 demonstrated that treatment with ACEIs and/or ARBs had no impact on the severity of the disease; moreover, it was associated with decreased mortality [83]. Another meta-analysis based on eighteen studies involving a total of 17,311 subjects demonstrated a 16% risk reduction in the composite outcome (death, admission to intensive care unit, mechanical ventilation requirement or progression to severe or critical pneumonia) in SARS-CoV-2-infected hypertensive patients treated with ACEIs or ARBs compared to no treatment or treatment with other substances than ACEIs/ARBs [84]. The most recent meta-analysis of 25 observational studies demonstrated a neutral effect of ACEI/ARB treatment on SARS-CoV-2 infection, disease severity, hospital/intensive care unit (ICU) admission or SARS-CoV-2-related death; however, the subgroup analysis revealed a decreased risk of critical illness and death in Asian populations, and an increased risk for ICU admission or death in North America and Europe, respectively [85].

Several theories aiming to explain the effect of RAS inhibiting treatment on SARS-CoV-2 infection at cellular and/or subcellular levels have been proposed [25,86,87]. However, the physiological and pathological roles of the majority of the involved molecules remain only partly understood and the complexity of their interactions raises a number of questions requiring further investigation.

Firstly, the impact of ACEIs and ARBs on ACE2 levels and activity has yet to be fully elucidated, since the available data are discrepant. For example, treatment with lisinopril in an animal experiment did not affect renal ACE2 activity [88]. AT1R blockade was associated with enhanced cardiac ACE2 expression and activity, whereas the co-administration of lisinopril and losartan led only to an increase in ACE2 activity but not to an elevation of mRNA expression [89]. Enalapril prevented a decline in ACE2 expression and activity after experimental myocardial infarction [90]; however, in a similar model, ramipril alone or in combination with valsartan failed to increase cardiac ACE2 [91]. In tissue samples from gastrointestinal endoscopy, ACE2 mRNA was increased in patients treated with ACE inhibitors but not with ARBs [92]. A Japanese cohort study involving 617 patients detected elevated urinary levels of ACE2 exclusively in patients treated with olmesartan, but not with other ARBs or ACEIs [93]. In a recent study, treatment with ACEIs or ARBs failed to increase plasma levels of ACE2 in two independent cohorts of heart failure patients [60].

These unequivocal and confusing findings indicate that the effect of antihypertensive treatment on ACE2 may vary depending on the drug and/or clinical conditions. ACEIs and ARBs have distinct mechanisms of actions and influence the components of the RAS in different ways. Treatment with ACEIs is associated with an accumulation of Ang I, Ang 1–7 and bradykinin, whereas ARBs do not influence the activity of bradykinin [26,94]. The effects on the systemic and local RAS are also diverse. For example, treatment with ARBs is related to strongly elevated plasma levels of Ang II [94]; however, renal or brain Ang II activities were shown to be attenuated during the administration of ARBs [95,96].

Similarly, there is a lack of information about the effect of antihypertensive agents on soluble and membrane-anchored ACE2. It is not obvious if ever or to what extent treatment with RAS inhibiting drugs affects ACE2 gene expression or the release of sACE2 from the cell membrane. Increased levels of Ang II in the case of treatment with ARBs might stimulate ACE2 shedding. On one hand, sACE2 is still able to catalyze the conversion of Ang II to Ang 1–7 as well as to bind coronaviruses, thus blocking their internalization by the target cells [49]. On the other hand, Ang II-induced shedding of ACE2 is associated with the activation of the ADAM17-mediated pathway [52,54], resulting in enhanced intracellular TNF-α-signaling, which contributes to tissue damage [56,57,97]. These unresolved questions, resulting in contradictory hypotheses, indicate that the modulation of the RAS by ACEIs and ARBs is complex and tissue specific. The clinical impact of ACEIs and ARBs may be settled by the ongoing prospective clinical trials [98].

## 8. Other Non-Classical RAS Components

Although ACE2 seems to be the key regulator in acute lung injury and ARDS, other non-classical RAS-associated molecules, such as alamandine, Ang 1–9, N-acetyl-seryl-aspartyl-lysyl-proline (Ac-SDKP) and the related alternative metabolic pathways also deserve attention. Since these molecules were shown to modulate inflammatory response in several organs, their potential interference with immune processes in the lungs should be considered.

Alamandine, a heptapeptide differing from Ang 1–7 in only one amino acid residue, is generated from Ang II metabolite Ang A or directly from Ang 1–7 by the catalytic action of ACE2 [11]. Alamandine attenuated neutrophil degranulation and induced the anti-inflammatory reprogramming of macrophages under inflammatory conditions in vitro as well as in vivo in mice [99,100] and improved cardiac function in LPS-induced sepsis by attenuating inflammatory reaction [101]. Oral administration of alamandine was associated with reduced expression of pro-inflammatory genes CCL2, TNF-α and IL-1β in the aorta in the mice model of transverse aortic constriction [102]. Presumably, the downregulation of ACE2 in acute lung injury may abrogate the cleavage of Ang A, which instead of being converted to the anti-inflammatory compound alamandine, can react directly with AT1R, contributing to pro-inflammatory and pro-fibrotic effects. It is thus reasonable to suppose that the potential protective effect of ACE2 in respiratory pathologies could in part be mediated by enhanced conversion of Ang A to alamandine.

Ang 1–9, another active metabolite generated in an alternative pathway from Ang I, can be further converted to Ang 1–7 or act directly on the AT2R [103]. Based on current knowledge, the molecule is considered as anti-inflammatory and anti-fibrotic. The administration of Ang 1–9 led to decreased ACE activity and reduced Ang II formation in the hearts of diabetic rats [104]. In the rat DOCA-salt model of hypertension, Ang 1–9 attenuated inflammation and protected against organ fibrosis in the heart, aorta and kidney [105]. In experimental pulmonary hypertension in rats, Ang 1–9 reduced plasma levels of inflammatory cytokines, such as TNF-α, MCP-1, IL-1β and IL-6, and reversed changes in the expression of apoptosis-related proteins, such as Bax, Bcl-2, Caspase-3 and -9 in the lungs [106].

Ac-SDKP, an alternative substrate for ACE, has been investigated in several pathologies regarding its antifibrotic, antiproliferative and anti-inflammatory properties [10]. Ac-SDKP was shown to reverse inflammation and fibrosis in the heart [107,108], aorta [109], kidney [110], liver [111] and lungs [112,113]. In in vitro assays, Ac-SDKP suppressed silicon dioxide-induced apoptosis in human alveolar type II epithelial cells (A549) [114]. Similarly, Ac-SDKP attenuated TGF-β1-induced epithelial-mesenchymal transition in A549 cells [113]. Additionally, Ac-SDKP in mice exposed to bleomycin, attenuated pulmonary edema, leukocyte infiltration and expression of IL-17 and TGF-β, reduced collagen content in the lungs and even improved survival [112]. Importantly, it even reversed already established pulmonary fibrosis [112]. Thus, Ac-SDKP may play a beneficial role in preventing chronic complications of acute lung injury.

Due to their anti-inflammatory and antifibrotic properties, certain non-classical RAS molecules other than ACE2 might represent a potential therapeutic target as well. However, their role in the pathogenesis of acute lung injury and ARDS as well as the possibility of their therapeutic modulation have yet to be elucidated.

## 9. Perspectives for Drug Development

Considering the unfavorable prognosis of ARDS and the lack of effective treatment [2,4], there is an urgent need for new therapeutic options. RAS modulating therapies might target several processes involved in the pathogenesis of ARDS (Table 2), such as excessive inflammation, increased vascular permeability as well as fibrotic remodeling of the lungs. In addition, in the context of the ongoing pandemic, agent-specific strategies aimed at eliminating the cell entry and/or replication of the pathogen are also of considerable importance.

The experimental administration of Ang 1–7 in several models of acute lung injury showed some protective potential. In a two-hit ARDS model (acid- and ventilation-induced injury) in rats, the administration of Ang 1–7 improved blood oxygen saturation, reduced the number of inflammatory cells in bronchoalveolar lavage fluid and reduced fibrosis in later stages of ARDS [36]. Improved oxygenation and alleviated inflammation related to Ang 1–7 treatment were also documented in LPS-induced lung injury in mechanically ventilated rats [115]. Moreover, Ang 1–7 suppressed LPS-induced AT1R mRNA expression in Sprague Dawley rats [30].

The administration of recombinant human ACE2 (rhACE2) could be another hopeful strategy [34,35,116]. This approach was associated with improved pulmonary function, alleviated inflammation as well as attenuated fibrotic remodeling in the lungs in animal experiments [34,35]. In the case of SARS-CoV or SARS-CoV-2 infection, increased sACE2 availability could bind and inactivate the virus, resulting in the reduced entry of the virus into the cells through the membrane-bound ACE2 [115]. The pharmacological properties of rhACE2 have already been studied on healthy volunteers as well as ARDS patients and the intravenous administration of the molecule was shown to be well tolerated without any relevant cardiovascular side effects [117,118].

ACE2 inhibition represents an alternative approach proposed to inhibit the entry of SARS-CoV-2 into the target cells [116]. However, this treatment could presumably also suppress Ang II conversion to Ang 1–7 by ACE2 and enhance the activity of the pro-inflammatory RAS axis. On the other hand, it has been suggested that treatment with a putatively competitive ACE2 agonist with higher receptor affinity compared to SARS-CoV or SARS-CoV-2 might block the internalization of the virus and stimulate ACE2/Ang 1–7-mediated signaling [25]. In addition, it seems logical to suppose that the stimulation of the ACE2/Ang 1–7 axis, e.g., by Ang 1–7, rhACE2 or ACE2 agonists in co-treatment with established agents inhibiting ACE or AT1R, could offer additional protection compared to monotherapy.

## 10. Conclusions

The role of the RAS is much more complex than vascular tone regulation or cardiac remodeling modulation; still, its effects beyond the cardiovascular system are less understood. Recent data suggesting that the RAS might be one of the principal regulatory systems controlling the immune response in acute lung injury open new horizons for further research in several directions. In the course of ARDS, two mechanisms contribute to organ damage: (1) the direct harmful effect of etiological factors (e.g., microbial agents, toxins etc.) causing an initial injury, and (2) immune-induced damage due to exaggerated inflammatory response. The biological functions of RAS-associated molecules, at least in relation to pulmonary tissue damage, should not be viewed as black or white. Their roles change dynamically during the development of acute lung injury to ensure, on one hand, an adequate immune defense and, on the other hand, pulmonary tissue restoration once the damaging factor has been eliminated.

The dominance of the classical ACE/AngII/AT1R pathway seems to be desirable in protecting the tissue against the initial injury caused by the etiological agent itself. In later phases, increased ACE2 activity presumably attenuates the immune response and protects against exaggerated inflammation associated with cytokine storm, profound tissue alterations and the fibrotic remodeling of the lungs. This increase in ACE2 activity might be decisive for tissue repair. Several pathogens have been shown to decrease the expression and/or activity of ACE2, which could lead to increased bioavailability of Ang II, DABK or Ang A as well as decreased levels of certain counterregulatory molecules, such as Ang 1–7 or alamandine. The disrupted equilibrium within the RAS could at least partly explain the mechanisms deteriorating and complicating the course of acute lung injury. The pilot experimental data suggest that targeting RAS-associated molecules might represent a novel and promising approach in ARDS therapy. However, numerous questions remain unresolved and the following research directions should be encouraged:

— First, the roles of membrane-bound and soluble ACE2 in the respiratory tract as well as the biological significance of ACE2-shedding under physiological and pathological conditions should be elucidated.
— The interaction mechanisms of particular infectious agents with the RAS, especially ACE2, are of importance for specific therapeutic approaches.
— Besides ACE2, other non-classical components of the RAS could also play a significant role in the pathogenesis of acute lung injury and ARDS.
— The biological impact of established RAS modulating therapies (ACEIs or ARBs) on RAS molecules should be disclosed in ongoing clinical trials.

A more profound understanding of the RAS role in the pathogenesis of acute lung injury and ARDS could shed more light on the therapeutic approach to the acute respiratory distress syndrome.

## Figures and Tables

**Table 1:** Effect of pharmacological inhibition of the renin–angiotensin system on respiratory pathologies. ACEIs—angiotensin-converting enzyme inhibitors; ARBs—angiotensin II-receptor type 1 (AT1R) blockers; COPD—chronic obstructive pulmonary disease; ICU—intensive care unit; T2D—type 2 diabetes mellitus.

Study Design	Subjects	Outcome	Ref.
Retrospective cohort study	254,485 patients > 65 y.o. newly prescribed antihypertensive drugs	↓ risk of hospitalization with pneumonia within 90 days following treatment initiation with ACEIs/ARBs vs. other antihypertensive drugs	[74]
Retrospective nested case–control study	375 COPD patients	↓ risk of pneumonia	[75]
Retrospective comparative study	12,452 patients newly prescribed ACEIs/ARBs within 90 days after diagnosis of COPD	↓ risk of pneumonia, severe pneumonia and ↓ mortality in ARBs group vs. ACEIs	[76]
Retrospective cohort study	215,225 patients	Improved infectious (influenza, pneumonia), inflammatory (COPD) and structural outcomes in ACEIs/ARBs vs. other treatment	[77]
Retrospective case–control study	182 ARDS patients	↑ duration of mechanical ventilation and ICU stay in ACEIs/ARBs group ↑ survival in ACEIs/ARBs group	[78]
Cox regression longitudinal observational study	1482 T2D patients	↓ risk of pneumonia/influenza	[79]

**Table 2:** Effect of angiotensin 1–7 and angiotensin-converting enzyme 2 in animal models of acute lung injury (ALI)/acute respiratory distress syndrome (ARDS).

Treatment	Experimental Model	Effect of Treatment	Ref.
Ang 1–7 LD: 0.27 μg/kg/h i.v. HD: 60 μg/kg/h i.v. Late-ARDS-study: 300 μg/kg/d i.v.	Sprague Dawley rats HCl- and ventilation-induced injury Late-ARDS- study: 2 weeks post-injury	Improved oxygenation (PaO_2_/FiO_2_) ↓ WBC in peripheral blood Late-ARDS: ↓ hydroxyproline content in lung HD: ↓ inflammatory cells in BAL	[36]
Ang 1–7 2.4 µg/kg/h i.v.	Sprague Dawley rats LPS- and ventilation-induced ARDS	↑ PaO_2_ ↑ACE2 and Ang 1–7 in BAL Normalized Ang-(1–7)/Ang II ratio$ ↓ CINC-3, TNF-α, GM-CSF in BAL	[115]
Ang-1–7 600 μg/kg/d i.p.	Sprague Dawley rats LPS-induced early pulmonary fibrosis	↓ lung injury and lung fibrose scores ↓ TGF-β in plasma ↓ Ang II in BAL Normalized E-cadherin and vimentin levels in lung ↓ AT1R mRNA expression in lung ↓ cell-membrane AT1R and ↑ MasR in lung ↓ LPS-induced phosphorylation of Src kinase	[30]
Ang 1–7 100 ng/kg/min s.c.	C57BL/6 mice LPS-induced ALI	↓ edema, bleeding, collagen and septal widening in lung ↓ TGF-β and p-SMAD2/3 in lung	[119]
ACE2 2 mg/kg i.p.	C57BL6 mice BLM-induced ALI	↑ survival, exercise capacity, lung function (dynamic compliance and elastance) ↓ collagen, α-SMA, TGF-β1, TNF- α in lung	[34]
ACE2 1.0 mg/kg i.v.	C57BL/6 mice LPS-induced ALI	↓ lung W/D ↓ PaCO_2_ and ↑ PaO_2_ ↓ lung histological score ↓ IL-1β, IL-6 and TNF-α in lung and in serum ↓ protein concentration and neutrophil count in BAL ↓ LPS-activated TLR4-signaling	[35]

ARDS—acute respiratory distress syndrome; ALI—acute lung injury; Ang 1–7—angiotensin 1–7; ACE2—angiotensin-converting enzyme 2; Ang II—angiotensin II; AT1R—angiotensin II-receptor type 1; i.v.—intravenously; i.p. intraperitoneally; s.c.—subcutaneously; HD—high dose; LD—low dose; BAL—bronchoalveolar lavage; HCl—hydrochloric acid; LPS—lipopolysaccharide; BLM—bleomycin; PaCO_2_—partial pressure of carbon dioxide; PaO_2_—partial pressure of oxygen; FiO_2_ –fraction of inspired oxygen; WBC—white blood cells; TNF-α—tumor necrosis factor α; TGF-β—transforming growth factor β; IL—interleukin; α-SMA—alpha smooth muscle actin; TLR—toll like receptor; CINC-3—cytokine-induced neutrophil chemoattractant 3; GM-CSF—granulocyte-macrophage colony-stimulating factor; W/D—weight/dry weight ratio.

## Abbreviations

α-SMA	α-smooth muscle actin
ACE	angiotensin converting enzyme
ACEIs	angiotensin converting enzyme inhibitors
Ac-SDKP	N-acetyl-seryl-aspartyl-lysyl-proline
ADAM17	tumor necrosis factor-α-converting enzyme (TACE)
ALI	acute lung injury
Ang	angiotensin
APA	aminopeptidase A
APM	aminopeptidase M
ARBs	angiotensin II type 1 receptor blockers
ARDS	acute respiratory distress syndrome
AT1R	angiotensin II type 1 receptor
AT2R	angiotensin II type 2 receptor
BAL	bronchoalveolar lavage
BLM	bleomycin
CATA	cathepsin A
CINC-3	cytokine-induced neutrophil chemoattractant 3
COPD	chronic obstructive pulmonary disease
CxA	carboxypeptidase A
CXCL5	C-X-C motif chemokine 5
DABK	des-Arg^9^-bradykinin
DOCA	deoxycorticosterone acetate
FiO_2_	fraction of inspired oxygen
GM-CSF	granulocyte-macrophage colony-stimulating factor
HD	high dose
ICU	intensive care unit
IL	interleukin
i.p.	intraperitoneally
i.v.	intravenously
KC	C-X-C motif chemokine 1
LD	low dose
LPS	lipopolysaccharide
mACE2	membrane-bound angiotensin converting enzyme 2
MasR	Mas receptor
MCP-1	monocyte chemoattractant protein-1
MIP2	macrophage inflammatory protein-2
MLDAD	mononuclear leukocyte-derived aspartate decarboxylase
MrgD	Mas-Related G-Protein Coupled Receptor D
NEP	neprilysin
PaCO_2_	partial pressure of carbon dioxide
PaO_2_	partial pressure of oxygen
POP	propyl oligopeptidase
PKC-δ	protein kinase C-δ
RAS	renin–angiotensin system
rhACE2	recombinant human angiotensin converting enzyme 2
sACE2	soluble angiotensin converting enzyme 2
s.c.	subcutaneously
T2D	type 2 diabetes mellitus
TACE	tumor necrosis factor-α-converting enzyme (ADAM17)
TGF-β1	transforming growth factor β1
TLR	toll like receptor
TMPRSS2	transmembrane protease serine 2
TNF-α	tumor necrosis factor-α
WBC	white blood cells
